# School difficulties in immigrant adolescent students and roles of socioeconomic factors, unhealthy behaviours, and physical and mental health

**DOI:** 10.1186/1471-2458-12-453

**Published:** 2012-06-19

**Authors:** Kénora Chau, Michèle Baumann, Bernard Kabuth, Nearkasen Chau

**Affiliations:** 1 Service of Pedopsychiatry, Hôpital d'Enfants de Nancy-Brabois, Université de Lorraine, Faculté de médecine, Vandoeuvre-lès-Nancy, France; 2 University of Luxembourg, INtegrative research unit on Social and Individual DEvelopment (INSIDE), Walferdange, Luxembourg; 3 INSERM, U669, Paris, F-75014, France; 4 Univ Paris-Sud and Univ Paris Descartes, UMR-S0669, Paris, France; 5 Inserm, U669, 8 rue du Breuil, F-54180, Heillecourt, France

**Keywords:** School difficulties, European immigrants, Non-European immigrants, Family characteristics, Socioeconomic status, Quality of life, Health-related behaviours

## Abstract

**Background:**

School is a multi-cultural setting where students need social, material, physical, and mental resources to attain school achievement. But they are often lacking, especially for immigrant students. In an early adolescence context, this study assessed risk for school difficulties among European and non-European immigrants and the roles of socioeconomic characteristics, physical health, psychological health, social relationships, living environment, and unhealthy behaviours.

**Methods:**

This cross-sectional study included 1,559 middle-school adolescents from north-eastern France, who completed a self-administered questionnaire including socioeconomic characteristics (gender, age, family structure, father’s occupation, and family income), WHO-Quality of life (measuring the four dimensions physical health, psychological health, social relationships, and living environment), unhealthy behaviours (last-30-day uses of tobacco, alcohol, cannabis, and other illicit drugs and no regular sports/physical activities), grade repetition, low school performance (<10/20), and school dropout ideation at 16 years. Data were analyzed using logistic models.

**Results:**

Grade repetition affected 14.8% of students, low school performance 8.2%, and school dropout ideation 3.9%. European immigrants had a higher risk for grade repetition only with a gender-age-adjusted odds ratio (OR) of 2.44, vs. French students. This odds ratio decreased to 1.76 (contribution 47%) with further adjustment for all confounders (family structure, father’s occupation, family income, physical health, psychological health, social relationships, living environment, and unhealthy behaviours). Non-European immigrants had a statistically higher risk for all grade repetition, low school performance, and school dropout ideation with ORs of 3.29, 3.02, and 3.42, respectively vs. French students. These odds ratios decreased to 1.76, 1.54, and 1.54, respectively (contributions 66%, 73%, and 78%) with further adjustment for all confounders.

**Conclusions:**

Compared with French students, European immigrant students were more affected only by grade repetition while non-European immigrant students by all grade repetition, low school performance, and school dropout ideation. The contribution of socioeconomic characteristics, physical health, psychological health, social relationships, living environment, and unhealthy behaviours was very high and much higher for non-European than for European immigrant students. Public policy should focus on these factors and services to reduce school difficulties.

## Background

With increasing migration the school is a multi-cultural setting, especially in the European Union. To attain school achievement students need social, material, physical, and mental resources, but they are often lacking. School difficulties can be described as an imbalance between learning conditions and the capacity of students to deal with them in their socioeconomic and cultural contexts. Grade repetition, low school performance, and school dropout ideation can occur early and then persist over time. They may lead to school dropout as a result of long-lasting lack of learning motivation, failure in expectation, and belief in school project. These schools issues may be more common among immigrant adolescents as their families are originating from less developed or developing countries with lower gross domestic product per inhabitant [1], and have lower education, socioeconomic status, and resources [2-4]. We need to assess the role of these factors with grade repetition, low school performance, and school dropout ideation that reflect different severities of school difficulties and to identify other potential factors such as unhealthy behaviours and physical and mental health, which are modifiable for prevention.

Families are the cornerstone of society and the main social environment for children. But families are changing. According to the OECD [5] many families now live in non-traditional arrangements, and there are more cohabitations, divorces, and remarriages. Children have fewer siblings and live more often with cohabiting, divorced/separated or single parents. More children are growing up in blended families of re-partnered adults. Over the past decade poverty in households with children is rising in nearly all OECD countries (12.7% across the OECD, and one in five children in Israel, Mexico, Turkey, the United States and Poland) [5]. Such poor living conditions are known to effect school achievement [2-4]. They generally start at an early age resulting in many students enter school with a variety of behavioral and emotional issues and lack of motivation for learning. The problem may be exacerbated among European immigrant adolescents and more among non-European immigrant adolescents because of lower parents’ education, socioeconomic status, and resources. It is thus important to evaluate their risk for school difficulties and the roles of family factors for school disengaging prevention.

School difficulties often appear early and may progressively generate school disengagement and absenteeism. They may lead to physical/psychological disorders, altered social relationships and supports, use of substances (tobacco, alcohol, cannabis, and other illicit drugs) to cope with difficulties, and lack of sports/physical activities. These factors may in turn alter the physical and mental capacities and may consequently reduce the chance for re-engaging in learning. Indeed, tobacco and alcohol consumption affect physical and psychomotor functions and cognitive performance [6-9]. There is a gateway from tobacco to cannabis and then to hard drugs [10,11], and cannabis use may exacerbate mental health difficulties [12]. Tobacco, alcohol, and cannabis use has been found to be associated with low school performance and leaving school without qualification [13,14]. Certain physical and psychological disorders such as sleep disorders, difficulties for working, concentration, tiredness, anxiety, and depression may impact both physical and mental capacities. Depression is known to affect work performance, cognitive ability, and workplace hazards monitoring [15-19] as well as memory and executive functions [15]. Adolescent mental disorders may need cares but, unfortunately, those of people with social/material deprivation are less likely to be treated [20-23]. In France, the percentage of people under poverty threshold (<60% of median income) was 7.5% in 2009; it reached 13.7% in individual aged 18–24, 20.8% in single parent families, and 43.8% in inactive mothers [24]. Four million individuals had not complementary health insurance in 2008 [25]. In the United States in 2010, there were 46.2 million people in poverty and 49.9 million people without health insurance coverage (after a regular increase in the rate since 1999) [26]. Physical and psychological disorders and unhealthy behaviours should thus affect more school difficulties (especially school dropout ideation). The knowledge of the roles of health disorders and unhealthy behaviours is thus needed for public policy aimed at limiting school difficulties. These factors are modifiable and may be targets for prevention. Despite that a number of studies have shown variations of school achievement across ethnic groups [2-4,14,27] no survey has investigated several measures of school difficulties among European and non-European immigrants and the roles of family characteristics, physical health, psychological health, social relationships, living environment, and unhealthy behaviours.

We focused on individuals in middle school students mostly under 16 years because school is compulsory in France until 16 years and many problems such as substance uses become persistent in late adolescence period (16–20 years) and all issues need to be solved sooner. Contrarily to other national studies we have participated [10,28,29] we chose to focus the present survey on the exhaustive population from a north-eastern French urban area so that the subjects are in the same socioeconomic context, free of variations across regions. The population studied was chosen because there is a clear social gradient while its health and health-related behaviours were close to those of the whole France (Appendix A).

In an early adolescence context, this school-population-based study aimed at to explore the risk for grade repetition, low school performance, and school dropout ideation at 16 years among European and non-European immigrants and the confounding roles of family characteristics, physical health, psychological health, social relationships, living environment, and unhealthy behaviours.

## Methods

### Procedure

The study population comprised all 1,666 students attending three middle schools chosen to reflect a social gradient in the Nancy urban area (410,000 inhabitants), the capital of Lorraine region (2,342,000 inhabitants) in north-eastern France. The population studied included all students from all the middle schools (two public and one private) in this area which included 63 classes. The investigation was approved by the Nancy-Metz regional education authority and the *Commission Nationale de l’Informatique et des Libertés* (national review board). Written informed consent was obtained from the respondents.

The study protocol included: an application to participate transmitted to parents/guardians via the students (April 2010), and data collection undertaken (from May to June 2010) using an anonymous self-administered questionnaire in the course of a class period (1 h), under the supervision of the research team and with the help of a teacher (when he/she wished, for surveillance with no influence on the survey). The completed questionnaire was put in a sealed envelope and then in a closed box by the subject. The questionnaire included questions on age, gender, father’s occupation, family structure, family income, the WHOQoL-Bref (measuring physical health, psychological health, social relationships, and environment) [30], last-30-day use and age at initiation for tobacco, alcohol, cannabis, and other illicit drugs, sports/physical activities (including those at school), school absenteeism during the school year, grade repetition during the life, last-trimester-school performance, and school dropout ideation at 16 years.

Grade repetition was assessed with the question ‘Have you repeated school year(s) at primary school and middle school?**’** (Never, at primary school, at middle school); multiple responses were possible. Grade repetition was defined as repeating at least one school-year Last-trimester-school performance was assessed with the question ‘What is your average school-mark for the last-trimester?**’** (7/20 or under, 8-9/20, 10-13/20, 14-15/20, 16/20 or more). Low school performance was defined as average school-mark below 10/20. School dropout ideation at 16 years was addressed in the question ’Do you wish to continue your study after middle school? (at university, vocational training, school dropout at 16 years).

Five father’s occupational categories were considered following the international classification of occupation (ISCO): professionals/managers/intermediate professionals; craftsmen, tradesmen, and heads of firm; service workers/clerks; manual workers/other occupations, and inactive people (unemployed and retirees). Professionals/managers/intermediate professionals were used as reference category. With regard to perceived income, subjects were asked whether the financial situation of their family was: comfortable or well off, earning just enough, coping but with difficulties, or getting into debt; low income was defined by coping, but with difficulties, or getting into debt [31,32].

Last-30-day use of tobacco, alcohol, cannabis, and other illicit drug uses were assessed with the questions [10,28,33,34]: ‘During the last 30 days did you smoke cigarettes?’ (none/1-4/5-9/10-19/20+ cigarettes/day), ‘During the last 30 days how many times have you had alcohol drinks (beer, cider, champagne, wine, aperitif, …?’ (none/1-5/6-9/10-29/30+), ‘During the last 30 days how many occasions have you used any form of cannabis?’ (none/1-5/6-9/10-29/30+), and ‘During the last 30 days how many occasions have you used any form of other illicit drugs (mushrooms, ecstasy, LSD, …)?’ (none/1-5/6-9/10-29/30+). These factors were dichotomized (at least once vs. none).

For WHOQoL-Bref the validated French version [35] was used. Past research has shown that the WHOQoL-Bref is a good, reliable, and valid cross-cultural scale appropriately used to measure the following domains in a multi-cultural setting: physical health, psychological health, social relationships, and environment [30]. It is the short-form of the World Health Organisation Quality of Life questionnaire. The World Health Organisation defines Quality of Life as “the individual’s perception of his/her position in life in the context of the culture and value systems in which he/she lives and in relation to his/her goals, expectations, standards, and concerns” [30]. In the European Union, quality of life is considered a high social and public health policy priority that reflects wider public concerns [36]. The WHOQoL-Bref domains were one-dimensional and reliable. Indeed, the Cronbach's alpha was 0.72 for the physical health domain, 0.70 for the psychological health domain, 0.62 for social relationships domain, and 0.78 for environment domain. They were similar to those of other studies [37-39].

Among the 1,666 subjects included in the population studied, 2 refused and 89 (5.3%) were absent when the data collection was carried out (for motive independent of the survey). In total 1,575 completed the questionnaire, of which 10 were of unknown gender or age, 9 were not completed appropriately, leaving 1,559 questionnaires (93.6%) for statistical analysis. The health and health-related behaviours of the sample were close to those of the whole France (Appendix A).

### Statistical analysis

The chi^2^ test or Fisher test was used to examine the relationships between each outcome variable (grade repetition, low school performance, and school dropout ideation) and nationality, gender, age, father’s occupation, family structure, WHOQoL-Bref domains (physical health, psychological health, social relationships, and environment), and unhealthy behaviours (use of tobacco, alcohol, cannabis, other illicit drugs, and lack of regular sports/physical activities). Then logistic models were used to yield gender-age-adjusted odds ratios. To study the associations between each outcome variable and ethnic group three logistic models were performed: a basic model (model 1) measured the associations adjusting for gender and age, and then with further adjustment for father’s occupation and family structure (model 2), and finally further adjusting for WHOQoL-Bref domains and unhealthy behaviours (model 3). The contribution of these factors to the explanation of ethnic group-outcome variable association was estimated by the change in the ORs i.e. explained fraction calculated by the formula: (OR_model 1_–OR_model 2_)/(OR_model 1_–1) or (OR_model 1_–OR_model 3_)/(OR_model 1_–1) [40]. Positive % values indicate reductions in ORs. The contribution was calculated only if the OR_model 1_ was significant. The analyses were performed using the Stata program (Texas: Stata Corporation 2007).

## Results

### Characteristics of subjects

They are shown in Table 1. French students represented 93.1%, European immigrants 3.5%, and Non-European immigrants 3.5%. Grade repetition affected 14.7%, low school performance 8.2%, and school dropout ideation 3.8%. During the last 30 days, 35.2% consumed alcohol, 11.2% tobacco, 5.6% cannabis, and 2.8% other illicit drugs. A relatively small proportion of students had no regular sports/physical activities (11.7%).

**Table 1:** Characteristics of subjects (n = 1,559)

	**%**
**Nationality**	
French	93.1
European immigrants	3.5
Non-European immigrants	3.5
**School difficulties**	
Grade repetition	14.7
Low school performance (<10/20)	8.2
School dropout ideation at 16 years	3.8
**Boys**	49.9
**Age (yr)**	
12 or under	38.7
13	23.9
14 or over	37.4
Mean (SD)	13.0 (1.3)
**Family structure**	
Intact	63.0
Parents divorced/separated and reconstructed family	25.1
Single parent and others	11.9
**Father’s occupation**	
Managers, professionals, and intermediate professionals	38.2
Craftsmen, tradesmen, and firm heads	20.1
Service workers and clerks	9.2
Manual workers and other occupations	25.0
Inactive people	7.5
Insufficient income	17.7
**WHOQoL-Bref (<25**^**th**^**percentile value)**	
Physical	23.2
Psychological	27.0
Social relationships	26.6
Environment	25.1
**Unhealthy behaviors**	
*Last-30-day substance use (at least once)*	
Tobacco	11.2
Alcohol	35.2
Cannabis	5.6
Other drugs	2.8
* Lack of regular sports/physical activities*	11.7

### Associations between nationality and various factors

Table 2 shows that significant differences were observed between French students, European immigrants, and non-European immigrants for family structure, father’s occupation, insufficient income, all WHOQoL-Bref domains, and for the consumption of tobacco, cannabis, and other illicit drugs. Immigrants initiated tobacco smoking earlier than French. Non-European immigrants initiated cannabis use earlier than the others. Absenteeism was more frequent among immigrants (especially for 15 days or more during the school year) except for that for a health problem.

**Table 2:** Associations between nationality and various factors: % or mean (SD)

	**French**	**European immigrants**	**Non-European immigrants**	**p-value**
*Number of subjects*	*1,451*	*54*	*54*	
Boys	50.0	44.4	51.8	NS
Age (yr) : Mean (SD)	13.0 (1.3)	12.8 (1.1)	13.4 (1.7)	NS
**Family structure**				<0.01
Intact	63.8	57.4	46.3	
Parents divorced/separated and reconstructed family	24.7	35.2	25.9	
Single parent and others	11.5	7.4	27.8	
**Father’s occupation**				<0.001
Managers, professionals, and intermediate professionals	39.5	22.2	18.5	
Craftsmen, tradesmen, and firm heads	19.9	24.1	22.2	
Service workers and clerks	9.2	5.6	13.0	
Manual workers and other occupations	24.5	38.9	24.1	
Inactive people	6.9	9.3	22.2	
**Insufficient income**	16.9	25.9	31.5	<0.01
**WHOQoL-Bref (<25**^**th**^**percentile value)**				
Physical	21.7	35.2	50.0	<0.001
Psychological	26.1	35.2	42.6	<0.01
Social relationships	25.6	25.9	53.7	<0.001
Environment	23.6	37.0	53.7	<0.001
**Unhealthy behaviours**				
*Last-30-day substance use (at least once)*				
Tobacco	10.5	16.7	24.1	<0.01
Alcohol	35.6	31.5	29.6	NS
Cannabis	5.1	9.3	14.8	<0.01
Other illicit drugs	2.3	7.4	11.1	<0.001
*Age at initiation for users (yr): Mean (SD)*				
Tobacco	12.1 (2.0)	11.6 (1.6)	10.8 (2.9)	<0.05
Alcohol	10.7 (2.2)	10.6 (2.3)	10.1 (3.0)	NS
Cannabis	12.9 (1.7)	12.8 (1.3)	10.7 (3.7)	<0.01
Other illicit drugs	12.4 (2.6)	12.7 (1.6)	11.0 (3.5)	NS
*Lack of regular sports/physical activities*	11.4	14.8	16.7	NS
**School absenteeism**				
Health problems	79.1	79.6	74.1	NS
Vacation	5.3	7.4	13.0	<0.05
Family problems	9.0	20.4	14.8	<0.01
Skipping school	5.6	11.1	22.2	<0.001
Others	11.8	6.3	27.8	<0.01
* Number of days during the school year*				<0.01
0	11.6	7.4	5.6	
1-7	70.7	61.1	64.8	
8-14	12.3	18.5	14.8	
> 14	5.4	13.0	14.9	

NS: non-significant (p > 0.05).

### Associations of school difficulties with various factors

As Table 3 shows, based on gender-age-adjusted odds ratios, European immigrants had a 2.44-fold higher risk for grade repetition compared to their French counterparts. Non-European immigrants had a 3-fold higher risk compared to their French counterparts for all grade repetition, low school performance, and school dropout ideation. Boys had a higher risk for school dropout ideation than girls. Older age was associated with an increased risk for grade repetition and low school performance. Non-intact families had a higher risk for all school difficulties. Compared to manager/professional/intermediate professional offspring, inactive offspring had the highest risk for all school difficulties, followed by manual worker offspring, service worker/clerk offspring, and craftsman/tradesman/firm head offspring (except for school dropout ideation for the last two groups). All four WHOQoL-Bref domains were related to grade repetition (except for social relationships) and the associations were stronger for low school performance and school dropout ideation. Lack of sports/physical activities and uses of all substance were positively associated with all school difficulties while alcohol use was negatively associated with grade repetition and was not associated with low school performance.

**Table 3:** Associations of school difficulties with various factors (n = 1,559): gender-age-adjusted odds ratio and 95% confidence interval

	**Grade repetition**		**Low school performance**		**School dropout ideation**	
	**OR**	**95% CI**	**OR**	**95% CI**	**OR**	**95% CI**
**Nationality**						
French						
European immigrants	2.44**	1.22-4.91	1.34	0.52-3.45	1.75	0.52-5.88
Non-European immigrants	3.29***	1.71-6.33	3.02**	1.52-6.02	3.42**	1.38-8.48
**Boys**	1.13	0.84-1.52	0.97	0.67-1.39	3.45***	1.88-6.34
**Age (yr)**	2.11***	1.86-2.40	1.42***	1.24-1.63	1.03	0.85-1.25
**Family structure**						
Intact						
Parents divorced/separated and reconstructed family	2.30***	1.64-3.23	3.10***	2.04-4.69	3.15***	1.71-5.78
Single parent and others	2.81***	1.83-4.31	3.49***	2.11-5.78	4.70***	2.37-9.32
**Father’s occupation**						
Managers, professionals, and intermediate professionals						
Craftsmen, tradesmen, and firm heads	1.77*	1.09-2.86	2.67**	1.39-5.11	1.82	0.79-4.18
Service workers and clerks	2.28**	1.28-4.07	2.85**	1.30-6.24	1.28	0.41-4.05
Manual workers and other occupations	3.49***	2.32-5.23	5.51***	3.14-9.67	3.08**	1.50-6.34
Inactive people	6.02***	3.56-10.18	6.85***	3.47-13.53	5.50***	2.34-12.95
**Insufficient income**	1.82***	1.28-2.59	1.77**	1.17-2.69	1.99*	1.11-3.57
**WHOQoL-Bref (<25**^**th**^**percentile value)**						
Physical	1.78***	1.29-2.47	3.77***	2.58-5.50	3.78***	2.21-6.49
Psychological	1.72***	1.25-2.37	3.09***	2.12-4.51	4.55***	2.66-7.79
Social relationships	1.37	0.99-1.88	1.48*	1.01-2.18	2.55***	1.50-4.31
Environment	3.01***	2.19-4.14	3.17***	2.18-4.60	3.40***	2.01-5.74
**Unhealthy behaviours**						
*Last-30-day substance use (at least once)*						
Tobacco	1.94***	1.31-2.87	5.33***	3.53-8.06	5.91***	3.33-10.49
Alcohol	0.68*	0.50-0.93	1.38	0.95-2.01	3.13***	1.80-5.43
Cannabis	1.78*	1.06-2.98	3.71***	2.17-6.33	5.91***	3.00-11.64
Other illicit drugs	2.61**	1.28-5.33	4.61***	2.30-9.25	10.11***	4.69-21.81
*Lack of regular sports/physical activities*	1.86**	1.23-2.82	2.21***	1.39-3.51	3.26***	1.80-5.88

*p < 0.05, **p < 0.01, ***p < 0.001.

### Associations of nationality with school difficulties and roles of father's occupation, family structure, WHOQoL-Bref domains, and unhealthy behaviours

As reported in Table 4, we showed that the gender-age-adjusted odds ratio of 2.44 for grade repetition for European immigrants decreased to 2.10 (24%) with further adjustment for father’s occupation and family structure, and to 1.76 (non-significant, 47%) with further adjustment for WHOQoL-Bref domains and unhealthy behaviours. The gender-age-adjusted odds ratios of 3.29, 3.02, and 3.42 for grade repetition, low school performance, and school dropout ideation, respectively, for non-European immigrants decreased by 39%, 40%, and 32%, respectively with further controlling for father’s occupation and family structure, and by 66%, 73%, and 78%, respectively with further controlling for WHOQoL-Bref domains and unhealthy behaviours.

**Table 4:** Associations of nationality with various factors (n = 1,559): odds ratio, 95% confidence interval, and contribution (%) of confounders

	**Grade repetition**			**Low school performance**			**School dropout ideation**		
	**OR**	**95% CI**	**%**^**a**^	**OR**	**95% CI**	**%**	**OR**	**95% CI**	**%**
**Odds ratio (OR**_**1**_**) adjusted for gender and age**									
French									
European immigrants	2.44**	1.22-4.91	100	1.34	0.52-3.45	-	1.75	0.52-5.88	-
Non-European immigrants	3.29***	1.71-6.33	100	3.02**	1.52-6.02	100	3.42**	1.38-8.48	100
**Odds ratio (OR**_**2**_**) with further adjustment for father’s occupation and family structure**									
French									
European immigrants	2.10* ^b^	1.03-4.31	24	1.04 ^b^	0.39-2.74	-	1.45 ^a^	0.42-4.98	-
Non-European immigrants	2.40* ^b^	1.20-4.81	39	2.22* ^b^	1.07-4.62	40	2.64* ^a^	1.02-6.83	32
**Odds ratio (OR**_**3**_**) with further adjustment for WHOQoL-Bref domains and unhealthy behaviours**									
French									
European immigrants	1.76	0.83-3.70	47	0.77	0.28-2.12	-	1.35	0.38-4.83	-
Non-European immigrants	1.78	0.86-3.68	66	1.54	0.68-3.51	73	1.54	0.49-4.78	78

*p < 0.05, **p < 0.01, ***p < 0.001.

Bold types: significant ORs.

^a^ % = Reduction (positive %) or increase (negative %) in OR computed with the following formula: (OR_1_ – OR_2_)/(OR_1_ – 1) or (OR_1_ – OR_3_)/(OR_1_ – 1); calculated for OR_1_ significant only.

^b^ Adding insufficient income to the model did not change the ORs.

## Discussion

This study demonstrates that, in the same socioeconomic area, European and non-European immigrants had higher school difficulties than their French counterparts and their risks were clearly different. European immigrants had a higher risk for grade repetition; 24% of the risk was explained by father’s occupation and family structure, and 47% by these family factors, WHOQoL-Bref domains, and unhealthy behaviours. Non-European immigrants had a higher risk for grade repetition, low school performance, and school dropout ideation; approximately 35% of the risks were explained by father’s occupation and family structure and 66%-78% by all confounders. This study is a part of a recent survey on health among French adolescents [41,42].

We focused on middle school students because a number of deleterious life events and health related issues started in the early adolescence period [10,43-45], and school difficulties need to be solved sooner. Many adolescents have poor living conditions [28,44,46] and their impact on mental health may be higher among the younger. Indeed, the first years at middle school correspond approximately to the mean age of onset of substance use, sleep disorders, suicidal ideation, involvement in violence, and violence victimization [28,47]. This is very troubling, especially because those issues generally persist over time.

In France, there were, in 2007, 2.56 million children aged less than 18 years in immigrant families. The birth country of the head of family was in Africa (1.37 million children, mainly in Algeria, Morocco, and Tunisia), Europe (642,302 children, mainly in Portugal, Spain, Italia, and United Kingdom), Asia (393,775 children, mainly in Turkey and Indochina), and America and Oceania (147,326 children) [48]. Therefore, most European immigrants are from developed countries while most non-European immigrants were from developing countries. Our study reveals that non-intact families were more represented among European immigrants and more markedly among non-European immigrants. Parents divorced/separated and reconstructed families were more represented among European immigrants while single parents were more represented among non-European immigrants. European immigrants were more likely to be from manual worker families while non-European immigrants were more likely to be from inactive ones. An important finding of our study is that family structure and father’s occupation explained 24% of the risk for grade repetition among European immigrants and about 35% of the risk for all grade repetition, low school performance, and school dropout ideation among non-European immigrants. It may be noted that adding insufficient income to the logistic models did not change the results. This should be explained by the strong relationships between family structure, father’s occupation, and insufficient income. Family structure and socioeconomic status appeared thus to be important barriers for school achievement among immigrants. European immigrants had thus a higher risk for school difficulties but they may not be persistent over time because they may disappear after a grade repetition. Non-European immigrants had much more school difficulties which may not be solved after a grade repetition and may lead to school dropout at 16 years with no qualification. A possible explanation is that their parents offer them less material conditions as well as lower engagement on their academic achievement which is known to be related to adolescents' academic engagement [49]. Their parents have too low educational levels to help the student with homework and to monitor their school results. The role of socioeconomic status, family structure, and families’ resources in school achievement is well known [2].

Another important finding of our study is that all physical health, psychological health, social relationships, and living environment (measured by WHOQoL-Bref domains) and unhealthy behaviours were strong factors for grade repetition, low school performance, and school dropout ideation. The present survey shows that adding these factors to the logistic models reduced the odds ratio for grade repetition by 47% for European immigrants while among non-European immigrants, it reduced the odds ratio by 66% for grade repetition, 73% for low school performance, and 78% for school dropout ideation. The contributions of confounders appeared here to increase with the severity of school difficulties. Our results call for several hypotheses:

(1) European immigrants had altered physical health, psychological health, and living environment compared to their French counterparts. These factors effect learning capacity leading to grade repetition, but the school difficulties may not be long lasting to result in persistent low school performance and school dropout ideation.

(2) Non-European immigrants had much more altered physical health, psychological health, social relationships, and living environment compared to their French and European immigrant counterparts. These factors are generally interdependent and persistent. We found that they were massive and may affect continuously learning capacity, motivation, leading to impressive school difficulties and especially to school dropout ideation. Because of these issues the students may think that learning is not worth for them or they were not able to do it over time.

(3) Current use of tobacco, cannabis, and other illicit drugs were more common among European immigrants and much more common among non-European immigrants compared to their French counterparts. Only non-European students initiated earlier use of all substances including alcohol. Our results confirm the relationships previously reported of tobacco, alcohol, and cannabis use with low school performance and leaving school without qualification [13,14]. Two school-based population studies in France and the United States demonstrated that early initiation of tobacco, alcohol, cannabis and other illicit drugs was associated with a higher risk for suicide ideation and suicide attempts [34,45]. As previously stated, tobacco and alcohol consumption may effect physical and mental performance [6-9] and cannabis use may exacerbate mental difficulties [12]. Positive attitude toward substance use is negatively associated with introversion, stability, and social conformity [50]. This would explain their strong associations with all outcomes variables with a trend according to their severity: the relationships between substance uses were high for grade repetition, clearly stronger for low school performance (with gender-age-adjusted odds ratios reaching 5.33), and much more for school dropout ideation (with gender-age adjusted odds ratios reaching 10). We conducted here a further analysis which showed that school dropout ideation was associated with a 6.98-fold higher risk for last-12-month suicide ideation and a 4.53-fold higher risk for lifetime suicide attempts (after controlling for gender and age).

Therefore, school may be seen by some immigrants and more especially by non-European immigrants as uninteresting, unchallenging, overwhelming or non-supportive. We found that absenteeism was more frequent among immigrants, especially for skipping school reaching 11.1% among European immigrants and 22.2% among non-European immigrants, vs. 5.6% among their native counterparts. This situation may lead the students concerned to develop negative attitudes (classes irrelevant, boring, just passing time, poor efforts, not able to connect with benefit in later life, attribution of responsibility for learning to teachers rather than to themselves, etc.) [51], and finally to avoidance of school and all it represents. It may be indicated that, in the same study, suicide behaviours were much more common among immigrants than among French students and the higher risk was strongly explained by family characteristics, school difficulties, unhealthy behaviours, and mental health [52].

This study demonstrates that grade repetition was strongly related to increasing age with a gender-adjusted odds ratio of 2.11. Age reflects in fact the duration of exposure to school difficulties. Low school performance was also related to age but with a lower gender-adjusted odds ratio of 1.42. This may reveal that school difficulties increased over time. It is very troubling that school dropout ideation was not related to age. This would mean that students who thought to dropout the school do it since a very young age. Whatever the initial causes, the longer the students have lived with them the more likely they have negative thoughts and re-engaging difficulties. Our results suggest that their living and learning conditions may be harmful, unsupportable or insurmountable giving to students little chance to see an improvement and expectations from year to year. It is not surprising that family structure, father’s occupation, physical health, psychological health, social relationships, living environment, and unhealthy behaviours contributed to 78% of the excess risk for school dropout ideation among non-European immigrants.

Regarding gender disparities, only school dropout ideation was more frequent among boys than girls with an age-adjusted odds ratio of 3.45. We conducted further analysis to explore the roles of confounders. Further adjustment for family structure and ethnic group did not change the odds ratio (3.56, 95% CI 1.93-6.58). Further adjustment for father’s occupation increased the odds ratio to 3.81 (95% CI 2.05-7.08, by 15%). Finally, taking into account all confounders increased the odds ratio to 4.78 (95% CI 2.45-9.33, by 54%). The gender difference for school dropout ideation was thus higher when controlling for all confounders. Boys had thus a 4.78-fold higher risk independently of all confounders. However, the risk for school dropout ideation was similar for non-European boys (age-adjusted odds ratio vs. French counterparts 4.82, p < 0.001, 95% CI 1.84-12.60) and girls (4.19, p = 0.067, 95% CI 0.89-19.60).

Some methodological aspects warrant comments. First, the study was based on self-reported data, but a self-administered anonymous questionnaire is widely used and arguably a good tool to study the living conditions, mental health, and unhealthy behaviours of adolescents [10,28,33,43,53]. A study on family factors and substance use among adolescents showed that self-report data were corroborated by independent teacher reports [54]. The European and non-European immigrants were well distributed in the 63 classes. The public school for each student was determined by his/her residence place and the precise class was attributed by the school. Second, as previously stated, most non-European immigrants are from Africa and Turkey. In France, it is not allowed to investigate specific ethnic groups. So we considered only three wide groups: French, European, and non-European immigrants which are clearly different in terms of socioeconomic and cultural characteristics. We focused this survey on the population from an urban area so that the subjects are in the same socioeconomic context, free of variations across regions. The interpretation of the results may be problematic if the native and immigrant students live in very different regions. Third, given the large number of statistical tests carried out, type I error may be a concern, but it has to be pointed out that most tests were significant at the 0.001 level, with very large odds ratios estimates. Although the socioeconomic conditions of the population studied are common through France and other developed countries, our findings cannot be generalized to other populations. They need to be confirmed by further studies.

Strengths of the study also deserve to be mentioned. The participation rate was high (93.6%). The data collection was made under the supervision of the research team and with the help of a teacher with no influence on the survey. The sample size allowed us to study European and non-European immigrants separately, which may be crucial because of their socioeconomic and cultural differences. All were made to guarantee the respondents’ anonymity. For this purpose, the questionnaire excluded the birthday, the birth place, and the residential town. Data collected and the respondents’ identification number do not allow the determination of school and the precise class. Some students needed however a confirmation about the anonymity when filling in the questionnaire. The quality of responses to the questionnaire was good. The different instruments were reliable and used in previous studies on wide samples of adolescents in France, the United Stated, and a number of other countries [10,28,33,34]. Grade repetition and low school performance are objective measures of school difficulties. Studying grade repetition, low school performance, and school dropout ideation at 16 years shed light on the levels of school difficulties and their risk patterns among European and non-European immigrants in early adolescence. It should be noted that the population studied was closed to the ESPAD (The European School Survey Project on Alcohol and Other Drugs) survey conducted on a large representative sample of school-based adolescents in France [28,33] for substance use, sleep disorders, asthma, depressive symptoms, suicide behaviours, victim of violence, and implication in violence measured using the same measures (Appendix A).

## Conclusions

Immigrant students had substantially higher school difficulties compared with their French counterparts. European immigrant students were more affected by grade repetition only while non-European immigrant students by all grade repetition, low school performance, and school dropout ideation. The higher risk was strongly confounded by family factors, physical health, psychological health, social relationships, living environment, and unhealthy behaviours. Public policy aiming at improving school achievement should focus on improvement of environment, living conditions, well-being, and monitoring physical and mental health and unhealthy behaviours as well as services to reduce school difficulties. Re-engaging disconnected students requires interventions to promote the positive perception of learning and learning environment, motivation, and benefit in later life. The impressive roles of confounders point out that all public policy should be made at an early age and based on a continuous physician-parent-school cooperation. This should promote social participation of adolescents when they are at school and also later at adulthood.

## Appendix A: Comparison between the study sample and France (ESPAD survey [28,33] (%)

**Table:** 

	**Study population (limited to <16 years**^**a**^**)(n = 1,524)**	**France <16 years**
		**(n = 8,367)**
Boys	49.9	48.9
Family structure		
Intact	63.2	74.7
Reconstructed	15.0	11.3
Single parent	16.4	11.7
Others	5.4	2.3
Obesity	10.6	6.9
Last-30-day substance use (at least once)		
Tobacco	10.7	13.6
Alcohol	34.7	34.6
Cannabis	5.1	5.5
Sleep disorders	32.6	29.0
Asthma	17.2	16.3
Depressive symptoms	13.1	9.8
Last-12-month suicidal ideation	11.6	9.1
Lifetime suicide attempts	9.6	7.2
Sexual abuse	3.4	1.9
Victim of violence	53.3	51.5
Implication in violence	59.1	64.7

^a^ 35 subjects were excluded due to age (≥16).

## Competing interests

The authors declare that they have no competing interest.

## Authors' contributions

KC conceived the survey, carried out the study and had the main responsibility for writing the manuscript. MB participated in conceiving the study and writing the manuscript. BK participated in conceiving the study and writing the manuscript. NC participated in conceiving the survey, statistical analyses and writing the manuscript. The authors read and approved the final manuscript.

## Pre-publication history

The pre-publication history for this paper can be accessed here:

http://www.biomedcentral.com/1471-2458/12/453/prepub
